# Solute–Vehicle–Skin Interactions and Their Contribution to Pharmacokinetics of Skin Delivery

**DOI:** 10.3390/pharmaceutics17060764

**Published:** 2025-06-10

**Authors:** Pronalis Tapfumaneyi, Khanh Phan, Yicheng Huang, Kewaree Sodsri, Sarika Namjoshi, Howard Maibach, Yousuf Mohammed

**Affiliations:** 1 Faculty of Pharmacy, Rhodes University, Grahamstown, Makhanda 6139, South Africa; pronalis.tapfumaneyi@yahoo.com; 2 Frazer Institute, Faculty of Health, Medicine and Behavioural Sciences, The University of Queensland, Brisbane, QLD 4102, Australia; k.phan@uq.edu.au (K.P.); k.sodsri@student.uq.edu.au (K.S.); s.namjoshi@uq.edu.au (S.N.); 3 School of Pharmacy and Pharmaceutical Sciences, The University of Queensland, Brisbane, QLD 4102, Australia; 4 University of California, San Francisco, CA 94115, USA; howard.maibach@ucsf.edu

**Keywords:** topical delivery, skin pharmacokinetics, partition coefficient, in-use conditions, metamorphosis of vehicle, skin–vehicle, skin–drug, vehicle–drug interactions, in silico models

## Abstract

Human skin provides an effective route of delivery for selected drugs. Topical penetration of molecules is largely attributed to passive diffusion, and the degree of penetration can be represented by in silico, in vitro, and ex vivo models. Percutaneous absorption of pharmaceutical ingredients is a delicate balance between the molecular properties of the drug, the skin properties of the patients, and the formulation properties. Understanding this interplay can aid in the development of products applied to the skin. The kinetics of percutaneous absorption and an understanding of the rate-limiting steps involved can facilitate the optimization of these systems and enhance the degree to which skin drug delivery can be achieved. Solute–vehicle, vehicle–skin, and solute–skin interactions contribute notably to product release as well as the rate of absorption and diffusion across skin layers. These interactions alter the degree of permeation by interfering with the skin barrier or solubility and thermodynamic activity of the active pharmaceutical ingredient. This article aims to provide a concise understanding of some of the factors involved in the skin absorption of topical products, i.e., the pharmacokinetics of percutaneous absorption as well as the solute–vehicle–skin interactions that determine the rate of release of products and the degree of drug diffusion across the skin.

## 1. Introduction

The skin has long been recognized as a crucial route for both local and systemic absorption of many active pharmaceutical ingredients (APIs) [1]. Over the past 50 years, topical and transdermal drug delivery systems have undergone significant evolution [2]. These advances are due to a progressively deepened understanding of drug–vehicle–skin interactions, related improvements in experimental methodologies and technology, and the Quality by Design (QbD) framework [3,4].

A key issue in skin drug delivery, which encompasses topical and transdermal, is the extent of drug absorption, specifically the ratio of the drug administered to the amount (approximately 30%) that successfully penetrates the stratum corneum (SC) [5,6]. Transdermal products, such as patches, are applied to the skin and rely on these interactions, especially the drug concentration gradient in the reservoir. While the site of action of transdermal products may/may not be the skin, their effect is more systemic.

Advancements in skin drug delivery technology, such as nanocrystals [7], microneedles [8], and medicated foams [9], have facilitated the design and optimization of these systems to understand the pharmacokinetics of percutaneous absorption. By targeting specific phases in percutaneous absorption, such as partitioning and diffusion (discussed in detail in the following sections), modifications can be made to enhance drug delivery to the skin [10].

Drug permeation across the skin primarily occurs via two main routes: (i) the transepidermal route, where drugs are transported via the SC, and (ii) the transappendageal route, where drugs are transported through sweat ducts and hair follicles [11]. For effective percutaneous absorption of drugs, the physicochemical properties of the permeate and the formulation matrix are critical and need to be considered during development [12]. These parameters include structural dimensions and the molecular mass of the permeant. Molecules < 500 Da are easier to permeate across the skin. Another crucial factor influencing percutaneous penetration is the log *p* value, an indicator of the permeant’s lipophilicity. The hydrophilic–lipophilic balance (HLB) can impact the ability of the molecule to penetrate the skin as it passes through the lipophilic SC into the aqueous phase (aqueous central compartment) of the circulation system. Surfactants included in the formulation matrix can influence the HLB balance, with high HLB surfactants enhancing transdermal delivery (absorption into bloodstream), whereas lower HLB surfactants enhancing topical delivery to deeper layers of the skin by increasing cutaneous residence time and drug deposition. The SC barrier, comprising of lipid and protein domains, is the rate-limiting step for drug permeation. The different physical and chemical characteristics of drugs, such as molecular weight and hydrophilic–lipophilic solute gradient, make the drug permeation process complex [12,13]. To circumvent the SC barrier, the drug must either traverse through corneocytes or permeate via hair follicles and sweat glands. Thus, the transepidermal and transappendageal pathways serve as drug-delivery pathways for targeted topical therapy [12]. The transepidermal route serves as a drug permeation pathway for low molecular weight solutes and lipophilic molecules, while the transappendageal route allows the passage of large hydrophilic molecules and colloid particles [14]. Melting point, the drug’s pKa (dissociation constant), and overall charge are the other major factors influencing the ability of drug molecules to dissolve and diffuse through the skin [12,15].

The transdermal permeation of molecules is largely attributed to passive diffusion, following a concentration gradient from high to low. Permeation profiles have been used to analyze the kinetics of skin permeation [16]. The porcine skin flap model is widely used to evaluate and simulate skin absorption [17], whilst in vitro [18] and in vivo [19] methods can be interpreted through quantitative analysis and pharmacokinetics. Mathematical models, such as those based on Fick’s diffusion law, have been used to express the degree of solute penetration at a steady state [20]:

(1) $$J=\frac{dq}{dt}=\frac{DPC_{v}}{h}$$

where J, the flux of the drug, is the rate of solute crossing the skin at steady state; q is the cumulative amount of solute per unit area of skin over time t; D is the diffusion coefficient; P is the partition coefficient between drug, vehicle, and skin; C_v_ is the concentration of the solute in the vehicle, and h is the membrane thickness.

Based on this equation, the flux of the drug (J) is directly related to the concentration gradient and the partition coefficient and inversely related to the membrane thickness [21].

While the discussion of percutaneous absorption and the fundamentals related to the process is extensive (as shown in Table 1), this article is focused on providing a concise review of some of the main factors involved in the skin absorption of topical products, i.e., the pharmacokinetics of percutaneous absorption as well as the possible interactions among solute/drug, vehicle, and skin that can determine the rate of release of products and the degree of drug diffusion across the skin. Emphasis is given on the mechanisms of permeation and not necessarily on the composition of the skin. It is noteworthy to mention that some of the information presented is related to works performed on in vitro, ex vivo, and mathematical models, which are not always a true and/or direct representative of what transpires in vivo. However, it does provide a good indication of some of the dynamics and implications certain factors might have on percutaneous absorption to help form a basis for in vivo–in vitro correlations (IVIVC).

This review provides key updates in the field in recent years. Table 2 compiles the physicochemical properties and characteristics of topical drugs approved (including those in the last 10 years). Additionally, through this review, we compare several case studies from the literature and explore the impact of drug–vehicle interactions on skin penetration. Several such attempts have been made; however, harmonization of study conditions remains a prerequisite for accurate comparisons of skin–vehicle, skin–drug, and vehicle–drug interactions.

## 2. Kinetics of Percutaneous Absorption

Much work has been performed to elucidate the kinetics of transdermal permeation, and it has been evidently demonstrated that the physicochemical properties of both the drug and vehicle play an important role in determining diffusion across the skin [22,23]. Variations exist in the absorption kinetics of intact skin compared to diseased, damaged, or removed skin [24], e.g., water-soluble or low molecular weight drugs have a higher rate of skin penetration in broken, diseased, or damaged skin compared to intact skin [25]. Even in intact skin, variations in the degree of permeation exist amongst different compounds, depending on the physicochemical properties of the skin and compounds, as well as the interactions involved [12]. Earlier research on skin permeability by researchers such as Scheuplein and Blank [25], Barr [26], Malkinson and Ferguson [27], Rothman [28], and Higuchi [21], amongst others, constitutes some of the fundamental material on percutaneous absorption, with evidence of skin permeability to compounds dating back to the 19th century.

**Table 1:** Selected recently published studies on percutaneous absorption, pharmacokinetics, and drug delivery systems.

Author	Title	Published Journal and Year	General Aims	Highlights	References
Roberts et al.	Topical drug delivery: history, percutaneous absorption, and product development	Advanced Drug Delivery Reviews, 2021	Describes the drug delivery of topical products from the perspective of their development over time, and how an active pharmaceutical ingredient (API) gets to its target site	Quantitative structure permeability relationships (QSPR) Molecular dynamics simulations Dermal physiologically based pharmacokinetics (PBPK) Topical product delivery behavior under ‘in use’ conditions and its in vivo response	[3]
Supe S, Takudage P.	Methods for evaluating penetration of drug into the skin: a review	Skin Research and Technology, 2021	Understand physiochemical characteristics impacting skin penetration of drug, as well as models employed for human skin permeation studies, and their advantages and disadvantages	Dermato-pharmacokinetics of different transdermal formulations Roles of different skin parts in percutaneous absorption and drug penetration Importance of evaluation methods and models in developing topical pharmaceutical formulations	[29]
Alkilani et al.	Beneath the skin: a review of current trends and future prospects of transdermal drug delivery systems	Pharmaceutics, 2022	Discussion on recent trends in transdermal drug delivery, and their advantages and disadvantages	Methods for improving drug permeability across the skin Hurdles restricting the application of transdermal drug delivery and future prospect	[30]
Feschuk et al.	Regional variation in percutaneous absorption in in vitro human models: a systematic review	Journal of Toxicology and Environmental Health, 2022	Outlines regional variation in percutaneous penetration in in vitro human models	Factors influence percutaneous penetrationImportance of ranking the susceptibility of different anatomical regions in: Transdermal drug delivery Decontamination protocols Pharmacologic/toxicologic judgments	[31]
Jin et al.	Metamorphosis of topical semisolid products—understanding the role of rheological properties in drug permeation under the “in use” condition	Pharmaceutics, 2023	Investigates metamorphosis of topical semisolid products—role of rheological properties in drug permeation under the “in use” condition	Effect of metamorphosis on rheological change during volatile solvent evaporation and on the permeability of API of topical semisolid formulations Role of metamorphotic events in understanding in vitro permeation profile	[32]
Chedik et al.	An update of skin permeability data based on a systematic review of recent research	Sci. Data, 2024	Update available skin permeability data by compiling recently published research	Inclusion and exclusion criteria selected to build the harmonized and reusable dataset of skin permeability Influence of different experimental parameters	[33]
Lee et al.	Advancements in skin-mediated drug delivery: mechanisms, techniques, and applications	Advanced Healthcare Materials, 2024	Provide an overview of skin-mediated drug delivery systems and techniques and mechanisms applied to enhance drug permeation across the skin	Skin-mediated drug delivery methods Mechanisms and techniques associated with skin-mediated drug delivery methods Recent advancements and limitations in the application of skin-mediated drug delivery	[34]

### 2.1. Partition from the Vehicle/Vehicle Release

**Figure 1:**
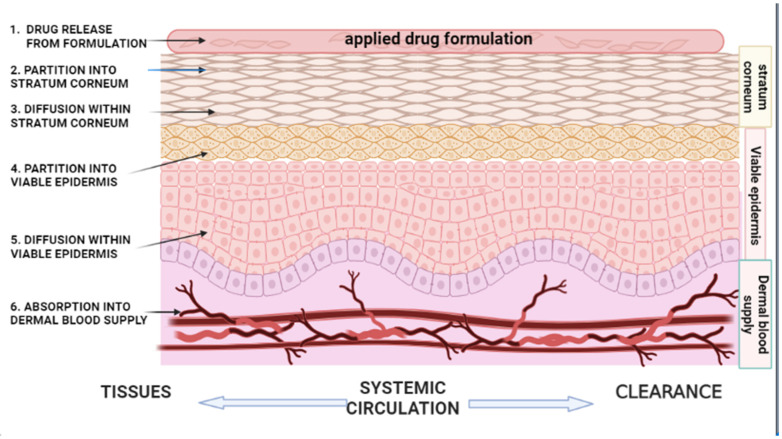
A schematic representation of the kinetics of percutaneous absorption. (1) Drug release/partition from the applied topical formulation vehicle; (2, 3) partition into and diffusion of the drug within the SC (rate-limiting steps for most drugs); (4, 5) partition into and diffusion of drug within the viable epidermis (rate-limiting step for highly lipophilic drugs); and (6) absorption of the drug into the dermal blood supply before going into the systemic circulation for distribution and clearance from the body (Figure created with BioRender.com).

The release of a drug/solute from a formulation vehicle into the SC (Figure 1) has been observed as the first step in percutaneous absorption [16], with the drug’s thermodynamic activity (a measure of the molecule’s tendency to escape from the formulation) being the limiting factor [20]. In a formulation vehicle, the thermodynamic activity of the API is a dynamic force for drug diffusion, where a higher thermodynamic activity can lead to a rise in the degree of dermal penetration [11,16]. The effect of a drug’s thermodynamic activity on percutaneous absorption will not be discussed in this review; however, Tapfumaneyi et al. [19] provide a comprehensive and updated analysis of the thermodynamic principle and its impact on the rate of skin permeation. The interactions between the drug and vehicle serve a crucial function in determining the degree of drug release from a formulation vehicle [35], which is largely dependent on the solubility of the drug as well as the partition of the drug between the vehicle and the skin [36]. This can be demonstrated by a water-soluble drug having to diffuse through oil in an oil-in-water emulsion that undergoes phase inversion to a water-in-oil emulsion before the drug can be absorbed through the skin [36].

The vasoconstrictor assay (VCA) has been a useful tool in determining the relation between the concentration of a drug—particularly corticosteroids—in a vehicle and the rate of drug release [37]. The VCA, also known as the human skin blanching assay, is an FDA-approved method used to measure the degree of topical corticosteroid percutaneous absorption, and was first described in 1962 by McKenzie and Stoughton [38]. It makes use of the skin-whitening side effect of topical corticosteroids, an effect first observed by Hollander et al. [39], which relates to the amount of drug that has penetrated the skin [40]. The VCA helps in establishing some of the differences in the degree of drug permeation (at the same concentration) of different vehicles [41,42]. For example, ointments have demonstrated their ability to create a barrier on the surface of the skin (owing to their occlusive nature), preventing water loss and enhancing hydration, which could vary the degree of permeation (depending on the drug) compared to other vehicles, such as creams, lotions, and solutions [19,43]. The VCA has also been useful in determining and ranking the potencies [19,42] of various topical corticosteroid formulation vehicles [41]. Studies by Zvidzayi et al. [42] and Rath et al. [41] demonstrated the effect of different formulation vehicles on drug permeation, with a 0.05% clobetasol cream demonstrating a greater degree of permeation compared to a slightly higher concentration of 0.1% clobetasol propionate propylene glycol solution. In the same studies, varying degrees of permeation were also observed from a similar concentration of 0.1% mometasone furoate cream and propylene glycol solution, with greater permeation observed from the topical application of the solution compared to the cream. The differences observed were attributed to the differences in formulation vehicles and associated vehicle properties, which either enhanced or impeded drug permeation and drug release from vehicles [41,42]. The VCA is thus a useful tool in determining the degree of drug release from topical formulation vehicles; however, the VCA only relates to topical corticosteroid products [42].

### 2.2. Partition into the Stratum Corneum (SC)

The drug’s solubility in both the formulation vehicle and lipid matrix of the SC determines the efficacy of topical products and their ability to circumvent the SC barrier [44,45]. The ability of drugs to diffuse across the SC barrier is dependent on the diffusivity of the drug and its solubility relative to the applied vehicle [46]. Since the SC is the rate-limiting step for drug transport across the membrane, following vehicle release, the drug has to partition into the SC (Figure 1) before proceeding to the dermis and epidermis [47]. This process involves dissolving in lipids on the surface of the SC, such as sebum and sweat [48,49], and binding to SC proteins [47]. This is dependent on the partition coefficient P (Equation (2)), which is the equilibrium of drug solubility in the SC relative to its solubility in the vehicle, represented mathematically as follows:

(2) $$P=\frac{Drug\;concentration\;(barrier)}{Drug\;concentration\;(vehicle)}$$

The partition coefficient regulates the extent of the driving force for absorption, i.e., the concentration gradient of the drug on the SC surface. An increase in lipid solubility increases the partition coefficient, leading to an increase in the degree of permeation [49]. Drugs that are lipophilic (hydrophobic) in nature and drug particles with a weight of less than 500 Daltons tend to partition into the lipid matrix more readily, with non-polar, hydrophilic drugs experiencing reduced permeability [50]. When the product of the partition coefficient and drug concentration in the vehicle (up to saturation solubility) is increased, the flux of the drug can be increased [46]. The presence of proteins determines the availability of the drug for diffusion through the SC [51], with the protein and lipid domains determining the degree of solute uptake depending on the drug’s lipophilicity [52]. To predict the lipid partition and protein binding properties of hydrophilic and hydrophobic drugs, equilibration experiments have been used to build quantitative structure–property relationships to demonstrate the drug’s chemical structure relative to its protein binding affinity and partition coefficient [47]. To determine the overall partition coefficient of hydrophilic and hydrophobic solutes, a two-phase model was developed to demonstrate the ratio of the drug’s concentration in the two phases, which is known as the partition coefficient [53]. Drug permeation across the SC is therefore dependent on the partition coefficient, the lipid and protein domains of the SC, as well as the drug’s chemical structure.

**Table 2:** Physicochemical properties † of topical drugs in currently marketed dermal products approved by the FDA (from Orange book [54]; data updated from [3,55] on 6 May 2025).

Drug Name	MW(Da)	MP (°C)	Log P	S_aq_ (mg/mL) at 25 °C	H_d_	H_a_
Acyclovir	225.2	255	−1.56	1.62, 2.50 (37 °C)	3	5
Adapalene	412.5	319–322	8.60	0.000004	1	3
Alclometasone dipropionate	520.2	212–216	3.94	0.14	1	7
Amcinonide	502.6	250–252	2.30	0.0077	1	8
Aminolevulinic acid hydrochloride	167.6	144–151	−2.80	173	3	4
Amlexanox	298.3	300	4.10	0.15	2	6
Ammonium lactate	107.1	91–94	−0.59	866	2	3
Amphotericin B	924.1	170	0.80	0.082	12	18
Avobenzone	310.4	84	4.51	0.0022	0	3
Azelaic acid	188.2	107	1.57	2.40	2	4
Bacitracin zinc	1422.7	250	−2.90	0.025	15	21
Benzoyl peroxide	242.2	103–106	3.46	0.0091	0	4
Benzyl alcohol	108.1	205	1.10	42.90	1	1
Benzyl benzoate	212.2	21, liquid	3.97	0.025	0	2
Betamethasone dipropionate	504.2	178	4.07	0.0046	1	5
Betamethasone valerate	476.6	184	3.60	0.0067	2	7
Bexarotene	348.5	230–231	6.90	0.00015	1	2
Bimatoprost	415.6	63–67	3.20	0.019	4	4
Brimonidine tartrate	442.2	207–208	1.27	0.15	6	9
Butenafine hydrochloride	353.9	208–210	5.67	0.00008	0	1
Calcipotriene	412.6	166–168	4.63	0.014	3	3
Capsaicin	305.4	65	3.04	0.029	2	3
Chlorhexidine gluconate	897.8	Liquid at 25 °C	2.71	0.026	18	16
Ciclopirox	207.3	144	2.30	1.41	1	2
Clindamycin phosphate	504.1	114	0.93	3.12	5	10
Clobetasol propionate	466.2	196	3.50	0.0039	1	6
Clocortolone pivalate	495.0	231–233	4.36	0.0010	1	6
Clotrimazole	344.1	148	0.50	0.00049	0	1
Crisaborole	251.1	129–135	3.24	0.023	1	4
Crotamiton	203.3	Liquid at 25 °C	2.16	0.018	0	1
Dapsone	248.3	175–177	0.97	0.28, 0.38 (37 °C)	2	4
Delgocitinib	310.4	N/A	1.29	0.552	1	5
Desonide	416.5	257–260	1.40	0.059	2	6
Desoximetasone	376.2	217	2.35	0.042	2	5
Diclofenac sodium	316.9	284	0.70	0.0048	1	3
Diflorasone diacetate	494.5	221–223	2.10	0.085	1	9
Docosanol	326.6	65–72	9.00	0.000000075	1	1
Doxepin hydrochloride	315.8	187–189	4.29	0.032	1	2
Dyclonine hydrochloride (Dyclopro)	325.9	175–176	4.66	0.049	1	3
Econazole nitrate	443.0	162	4.67	0.0015	1	5
Efinaconazole	348.4	86–89	3.70	0.32	1	6
Eflornithine hydrochloride	218.6	181–184	−2.19	50	4	6
Erythromycin	733.5	191	3.06	0.46	5	14
Fluocinolone acetonide	452.2	266–268	2.48	0.055	2	8
Fluocinonide	494.5	309	3.19	0.0047	1	9
Fluorouracil	130.0	280–282	−0.89	11.10 (22 °C)	2	3
Flurandrenolide	436.5	247–255	2.88	0.0011	2	7
Fluticasone propionate	500.6	261–273	3.38	0.011	1	9
Gentamicin sulfate	516.6	218–237	−3.10	100	8	14
Halcinonide	455.0	276–277	3.30	0.011	1	6
Halobetasol propionate	484.2	213–215	3.73	0.022	1	5
Hexachlorophene	405.8	164–165	7.54	0.14	2	2
Hydrocortisone	362.2	220	1.61	0.32	3	5
Hydrocortisone butyrate	432.6	210–214	3.21	0.014	2	6
Hydrocortisone valerate	446.6	217–220	3.62	0.0076	2	6
Hydroquinone	110.1	172.3	0.59	72	2	2
Imiquimod	240.3	292–294	2.70	6.25	1	3
Ingenol mebutate	430.5	153.5	3.12	0.0043	3	6
Ivermectin	875.1	155	5.83	0.0040	3	14
Ketoconazole	530.1	146	4.35	0.00029 (20 °C)	0	6
Lidocaine	234.2	69	2.44	4.10 (30 °C)	1	2
Lotilaner	596.8	N/A	5.81	N/A	2	4
Luliconazole	354.3	149–154	2.59	0.066	0	4
Mafenide acetate	246.3	177	−0.37	5.18	3	6
Mechlorethamine hydrochloride	192.5	108–110	0.91	33.40	1	1
Metronidazole	171.1	158–160	−0.02	11	1	4
Miconazole nitrate	479.1	159–163	3.26	0.026	1	5
Minoxidil	209.3	248	1.24	2.20	3	3
Mitomycin	334.3	360	−0.40	8.43	3	8
Mometasone furoate	521.4	218–220	3.90	0.011	1	6
Mupirocin	500.2	77–78	3.44	0.027	4	9
Naftifine hydrochloride	323.9	172–175	3.59	0.00023	1	1
Neomycin sulfate	712.7	187	−7.80	50	15	23
Nystatin	926.1	160	0.50	0.36	12	18
Octinoxate	290.4	Liquid at 25 °C	5.80	0.00045	0	3
Oxiconazole nitrate	492.1	137	3.80	0.0019	1	6
Oxybenzone	228.2	65.5	3.79	0.0037	1	3
Oxymetazoline hydrochloride	296.2	181–183	4.87	0.0014	3	2
Penciclovir	253.3	275–277	−1.10	7.45	4	5
Permethrin	391.3	34	6.50	cis isomer-0.00020, trans isomer-0.00013	0	3
Pimecrolimus	810.4	135–136	4.40	0.0015	2	11
Podofilox (Condylox)	414.4	228	2.01	0.15	1	8
Polymyxin B sulfate	1301.6	217–220	−0.74	0.074	20	29
Prednicarbate	488.6	114–116	2.92	0.0056	1	8
Prilocaine	220.2	37–38	2.11	0.54	2	2
Retapamulin	517.8	125–127	5.00	0.00039	1	6
Roflumilast	403.2	158	4.47	0.0062	1	6
Ruxolitinib	306.4	86	2.94	0.116	1	4
Sertaconazole nitrate	500.8	158–160	3.33	0.0064	1	6
Silver sulfadiazine	357.1	285	0.39	7.87	1	6
Spinosad	1287.7	112–123	4.00	0.0024	2	19
Sulconazole nitrate	460.8	130	3.21	0.0013	1	5
Sulfacetamide sodium	236.2	257	−0.96	50	1	5
Tacrolimus	804.0	126	3.30	0.000018	3	12
Tapinarof	254.3	140–142	4.25	0.0339	2	2
Tazarotene	351.5	103–105	3.38	0.00075	0	4
Terbinafine hydrochloride	327.9	204–208	3.30	0.00074	1	1
Tetracaine	264.4	41–45	3.51	0.56	1	4
Tretinoin	300.2	180–182	6.30	0.0048	1	2
Triamcinolone acetonide	434.2	293	2.53	0.08	2	7
Trifarotene	459.6	245	6.12	0.00095	2	5

† Physicochemical properties are acquired from PubChem (https://pubchem.ncbi.nlm.nih.gov/), ChemSpider (https://www.chemspider.com/), DrugBank (https://www.drugbank.ca/), Pastore et al. 2015 [54], Chemical Book (https://www.chemicalbook.com/), and product information sheets from manufacturers. MW, molecular weight; MP, melting point; Log P: log octanol–water partition coefficient; S_aq_: aqueous solubility of the unionized form; H_d_: hydrogen bond donor; H_a_: hydrogen bond acceptor.

### 2.3. Diffusion Within Stratum Corneum (SC)

Following the release of the API/drug from the formulation vehicle, the drug substance undergoes partitioning and diffusion through the skin from a high to low-activity site. In other words, API transport across the skin is dependent on the thermodynamic gradient [11], where an increase in the thermodynamic activity of the drug can increase the interfacial transport of the drug into the SC, resulting in increased drug concentration and higher drug permeation through the skin [56]. The SC can act as a depot, where the drug is released slowly into the body long after application (usually over a few days) [57]. The movement of the drug through the SC is represented by the diffusion coefficient D (Equation (1)), where the rate of drug penetration is directly related to the diffusion coefficient. The diffusion coefficient measures the degree of interaction and restriction of the SC with the mobility of the drug; thus, it is dependent on the characteristics of both the SC and the drug [10,49]. Factors that promote drug interaction with the SC affect the rate of drug diffusion [49]. For example, lipid and water-soluble substances have a greater degree of penetration compared to polar substances. When the water/lipid partition coefficient is close to the skin, permeability is optimal due to their interactions with the SC phospholipid bilayer [28]. The diffusion pattern of a drug may also be influenced by the physicochemical properties of the vehicle, which may interfere with the skin barrier and affect permeation [25,49]. This can be shown by the molecular size of a drug, which determines the resistance to movement, with larger molecules having greater frictional resistance to movement; for example, corticosteroid molecules are relatively large (such as Betamethasone Valerate (504 Daltons) and Mometasone Furoate (521 Daltons)) and therefore have poor movement within the SC barrier [49]. Drug diffusion within the SC is also affected by regional variations in the thickness of the SC, with areas with thicker skin, such as the palmar and soles, having different drug diffusion rates compared to thinner skin regions such as the arms, legs, and back [58].

**Figure 2:**
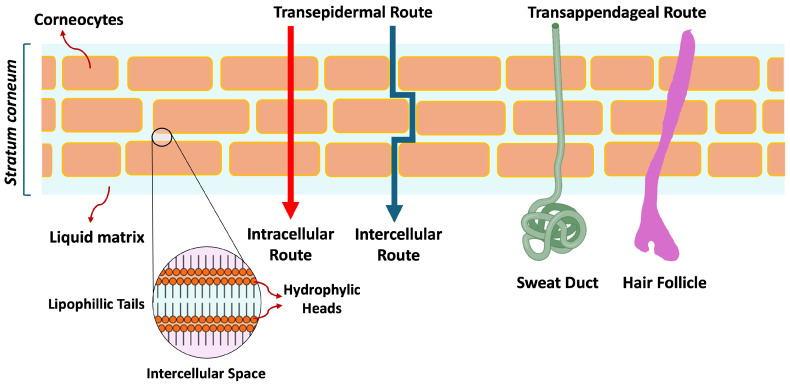
Drug permeation routes through the skin. The transepidermal route is divided into intercellular and intracellular/transcellular routes, with the transport of drugs occurring via the SC. The transappendageal route is divided into transglandular and transfollicular routes, transporting drugs via hair follicles and sweat ducts. Adapted and reproduced with permission from Ref [11]. Copyright 2023 Drug Discovery Today.

Drug diffusion into the skin occurs mainly via the transepidermal and transappendageal routes. The SC, as the primary barrier to skin permeation, can be modulated through interactions with topical drug delivery systems, thereby affecting the absorption of topical products [3]. The three transepidermal pathways for permeating the SC are intercellular, transcellular, and transappendageal (through hair follicles, sebaceous glands, and sweat glands) [59,60]. The transappendageal route is divided into transglandular and transfollicular routes (Figure 2). The intracellular route is considered to be a more direct route, responsible for drug absorption through the lipid structure of the SC [61,62]. The intercellular route, which is a more common drug delivery route, allows lipophilic and non-polar solute absorption between the cells (corneocytes) [29,63], and researchers [64,65] have argued that it is the main route of skin penetration. The passage of drug molecules between the cells helps circumvent the SC barrier [61]; however, the drug has to traverse both the lipophilic and hydrophilic structures within the SC, which limits the rate of absorption [66] owing to its tortuosity, resulting in a greater effective thickness compared to the SC [67]. The transappendageal route is for the absorption of drug molecules through hair follicles, sebaceous glands, and sweat glands [59,60]. The contribution to absorption via this route is considered to be small, as glands and hair follicles only account for 0.1% of the total human skin surface [68]. However, targeted follicular drug delivery has been demonstrated to be a relevant pathway for percutaneous drug delivery [69,70,71], with Tolentino et al. [72] demonstrating enhanced targeted drug delivery of clindamycin (entrapped in chitosan and hyaluronic acid nanoparticles) via the pilosebaceous glands compared to clindamycin commercial gel formulation. Their results showed that chitosan nanoparticles targeted 53%, and hyaluronic acid nanoparticles targeted 77% of the drug to the pilosebaceous glands, compared to 26% from the commercial gel [72]. Ban et al. [73] showed efficient delivery of myricetin lipid nanoparticles (designed to reduce excessive sweating) via sweat glands (transappendegeal pathway) by targeting sudor motor nerves located below the sweat glands, which control sweat gland activity. Their results showed a strong signal detection (using fluorescence microscopy- Leica TCS SP8 HyVolution confocal microscope, Leica Microsystems, Wetzlar, Germany) around hair follicles compared to intercellular spaces after the application of the lipid nanoparticles, suggesting drug delivery via the transappendageal pathway [73].

### 2.4. Partition into the Viable Epidermis

**Figure 3:**
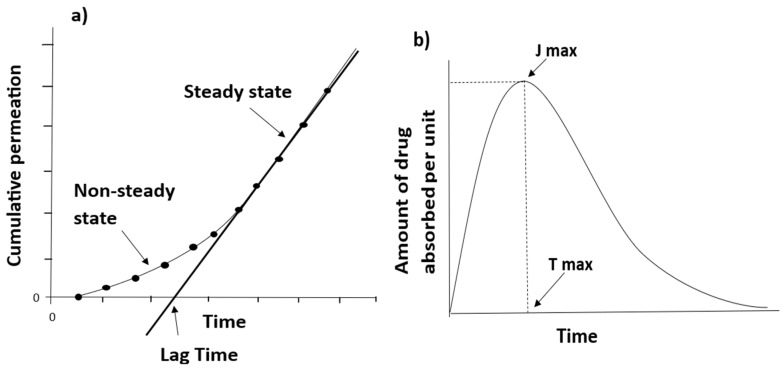
Typical permeation profile showing (**a**) infinite dosing initially at a non-steady state, followed by delayed drug absorption at a lag time before reaching steady-state diffusion; (**b**) finite dosing conditions with an increase in the flux of drug until reaching maximum value (J_max_), followed by a decrease in flux until reaching a plateau. Adapted and reproduced with permission from Ref [75], licensed under the CC BY-NC-ND license (https://creativecommons.org/licenses/by/4.0/) access on 13 November 2023. Copyright 2022 MDPI.

After diffusion through the SC, drug molecules enter the viable epidermis (Figure 1), where the rate-controlling step is the solubility ratio in both the SC layer and the viable epidermis. This phase begins before diffusion into the SC ends [48]. The epidermis and dermis are hydrophilic by nature [74]; hence, lipophilic molecules experience low mobility at this stage [48]. Fick’s first law represents the amount absorbed in the viable epidermis mathematically as follows [48]:

(3) Q=PACt

where Q is the amount of drug absorbed (g), A is the application area (cm^2^), t is the application time (h), C is the applied concentration (g/cm^3^), and P is the permeability coefficient (cm/h). Based on Equation (3), Q is directly proportional to P, C, A, and t. The driving force for the movement of molecules is the concentration difference on each side of the SC. After a short period, steady-state flux is reached during which the amount of the drug entering and leaving the SC are equal. The steady-state flux resumes provided the solute concentration in the applied preparation remains constant, which is considered infinite dosing (Figure 3a), and is usually observed in topical applications reapplied consistently [48,75]. Lag time is the time before reaching a steady state, where there is a delay in drug absorption into viable tissue [48].

However, for finite dosing, there is an initial increase in flux until it reaches a maximum value (J_max_) (Figure 3b), and as the drug concentration decreases, the concentration gradient also decreases, reaching a plateau [76]. This is usually shown by molecules with a higher SC affinity (highly absorbed) or for very small dose applications [48]. Usach et al. [75] evaluated the feasibility of nortriptyline (NT) gel formulation for transdermal administration and observed plasma levels of about 150 ng/mL between 8- and 30-h post-administration. They also observed the useful correlation between an estimated kinetic parameter (k_out_) (determined in vitro) and predicted in vivo plasma levels [75].

### 2.5. Diffusion Within the Viable Epidermis

The viable epidermis is composed of multiple skin layers made up of keratin-filled cells [77,78]. Transepidermal passage of molecules is governed by pharmacokinetic parameters and diffusion laws, as opposed to Fick’s laws that govern SC absorption [48]. The permeability and concentration of a drug in the viable epidermis are particularly important in instances when the SC is broken, damaged, or removed. Permeation through the SC becomes greater than the epidermis, and in such cases, the rate-limiting step for drug diffusion becomes the viable epidermis [79]. The dermal concentration provides an indication of the therapeutic drug quantity and resulting toxicity. Drug transport within the viable epidermis, i.e., epidermal and dermal layers, is often predicted using mathematical models [5]. Concentration in the viable skin can be modeled by assuming a homogenous membrane with isotropic diffusion represented mathematically as follows:

(4) $$\frac{\partial C_{vs}}{\partial t}=D_{vs}\frac{\partial^{2}C_{vs}}{\partial z^{2}}$$

where $C_{vs}$ is the concentration in the viable skin, z is the depth, and $D_{vs}$ is the diffusion coefficient. Equation (4) shows a direct correlation between the rate of drug penetration in the viable epidermis and the diffusion coefficient. This model provides useful information for concentration in the upper portion of the viable epidermis. Another model that can be used to determine viable epidermal concentration is a two-layer model, which separates the SC and viable epidermis into two layers to give a concentration-depth profile [79].

### 2.6. Absorption into the Dermal Blood Supply (Systemic Circulation)

Once the drug reaches the dermal layer, body fluid distributes it via the circulatory system to the rest of the body’s cells, after which the drug undergoes metabolism and elimination (Figure 1) [36]. The drug’s affinity to proteins and albumin, as well as the local blood flow, are important factors in systemic drug distribution [51]. Blood flow can be increased by vasodilation, which promotes the systemic distribution of the drug, whereas vasoconstrictive effects of drugs, such as corticosteroids, may decrease systemic absorption [80]. Some drugs reach systemic circulation via absorption from blood vessels in the subcutaneous tissue, e.g., opioids, whilst other drugs, such as lidocaine and capsaicin, act locally via receptors and ion channels [81]. Analgesics tend to target the nociceptive pathway and minimize plasma absorption of drugs, hence minimizing the potential for side effects [82]. Although the degree of systemic exposure from topical products is minimal compared to other routes of drug delivery, such as the oral route, there is still potential for systemic adverse effects from topical and transdermal drug delivery. For example, if misused, topical corticosteroids have the potential to cause systemic adverse effects such as Cushing’s syndrome [83].

The concentration of the drug after application is usually highest near the application surface of the skin and lowest in the dermis [84]. For drugs such as corticoids, a small amount of the applied dose permeates the skin, with most of the drug left on the skin, whereas occluded doses form a reservoir in the skin that prolongs drug absorption. Compounds such as benzoic acid are considered to be highly absorbed, with the majority of the drug excreted via urine [84]. The amount of drug in the body is often determined using the rate of urinary excretion [36]. Tomalik-Scharte et al. [85] used the rate of urinary excretion in determining the degree of systemic exposure to permethrin (used in the treatment of scabies) after topical administration to the hair of human participants. Their results demonstrated the extent of systemic exposure to permethrin, with low doses of the drug detected after gas chromatography/electron capture analysis of urine samples [85]. A good correlation between in vivo urinary excretion data and data from an in vitro permeation test for ethyl and glycol salicylates was demonstrated by Liu et al. [86], who used skin diffusion-based pharmacokinetic models to predict urinary excretion-time profiles from the in vitro permeation test data using a Laplace convolution procedure. Their results demonstrated how urinary excretion data can be predicted from in vitro skin permeation tests [86], signifying the usefulness of urinary excretion data in determining the degree of drug permeation following topical application of salicylate esters.

## 3. Solute–Vehicle–Skin Interactions and Percutaneous Absorption

### 3.1. Vehicle–Drug Interactions

The formulation of a transdermal product is aimed at ensuring that there is optimal drug delivery to the target site [87]. Drug release from the vehicle formulation is the first step in percutaneous absorption, and the rate of drug release varies depending on the formulation [88]. Interactions between the vehicle and drug can control the drug release profile across the skin barrier and may affect the availability of solute [89,90]. An example is the drug release rate from a solution vs. a suspension, given by Equations (5) and (6), respectively [91]:

(5) $$\frac{dQ}{dt}=C_{0}{\left(\frac{D}{\pi t}\right)}^{0.5}\;\mathrm{for}\;Q\;<\;30\%$$

(6) $$\frac{dQ}{dt}={\left(\frac{C_{0}C_{v}D}{2t}\right)}^{0.5}$$

where dQ/dt is the rate of drug released to the surface of the skin (per unit area), D is the drug diffusivity in the formulation vehicle, C_0_ and C_v_ are the initial concentration of drug and the drug solubility in the formulation vehicle, respectively, and t is the time when the amount of drug released is determined.

Based on Equations (5) and (6), the rate of release is dependent on the concentration of the drug, the diffusion coefficient (for both suspensions and solutions), and the solubility of the drug (for suspensions) in the vehicle. Essentially, for a given drug concentration, the rate of release is greatest for a formulation vehicle where the drug is completely soluble, and an increase in concentration increases the rate of release. For solutions, Equation (5) is only valid when the amount of drug released (Q) is less than 30% of the total drug, as the kinetics of drug release may significantly differ once a certain proportion of drug has been released [91]. In an ideal system (no skin–drug–vehicle interactions), the flux of a drug is directly proportional to vehicle–solute concentration; however, solutes sometimes interact with the vehicle, which may alter the solute concentration and affect the permeability coefficient (K_p_) [92]. The permeability coefficient measures the speed of transport across a membrane and is mathematically expressed as follows [93]:

(7) $$K_{p}=\frac{DP}{h}$$

Using Fick’s diffusion law in Equation (1), Equation (7) can be simplified as follows [92]:

(8) $$J=K_{p}C_{v}$$

Equation (8) suggests a direct relationship between flux, solute concentration, and skin permeability [92]. The vehicle can have a significant impact on the pharmacokinetic behavior of drugs and their ability to penetrate the skin [94]. During drug diffusion into the skin, only the soluble drug can penetrate the skin, and this is largely dependent on the thermodynamic activity of the drug in the vehicle [95]. Low solute solubility in the vehicle, associated with a low thermodynamic activity of the drug tends to reduce flux [96]. At saturation solubility in the vehicle, the drug is believed to have its maximum thermodynamic activity, which increases the membrane–vehicle partition coefficient and, hence, increases the degree of permeation. Therefore, a drug that is partially soluble in a vehicle may also equally experience partial release from the formulation vehicle, which may, in turn, affect the bioavailability of the drug [97,98]. Thus, permeation is optimal at the maximum solubility concentration in the vehicle [97]

The partition coefficient between the skin and the vehicle demonstrates the degree of affinity between the drug and the vehicle. The higher the partition coefficient, the lower the vehicle’s affinity for the drug; hence, the drug will have a greater tendency to leave the vehicle, which promotes permeation [98]. Colo et al. [99] investigated the influence of solvent power and microscopic viscosity of a vehicle on the release of benzocaine from a hydrogel to establish the effects of drug solubility and diffusivity in the vehicle on percutaneous absorption. The results suggested that changes in the solubility of the drug in the vehicle were directly related to the rate of release, whereas the microscopic viscosity of the gels was inversely related to release [99].

The modeling of skin absorption has been explored since the 1990s, and Quantitative Structure–Permeability Relationships (QSPRs) models have been designed to predict the degree of drug permeation based on its molecular structure [100]. Experimental data and statistical methods have been utilized in identifying the key determinants of permeability—i.e., molecular size and hydrophobicity (measured as the logarithm of the octanol–water partition coefficient) [101]. Experimental data from in vitro assays under steady-state conditions are often used as the basis for QSPR models, and Fick’s Law can be used to explain drug permeation at steady state [100]. QSPR models have been used as a method to quantify differences in flux between various drugs relative to the physicochemical properties of the vehicle. However, the development of robust and predictive QSPR models depends on the consistency and reliability of experimental data produced under standardized conditions, i.e., temperature, drug concentration, and receptor medium [102]. Based on a QSPR model, Najib et al. [102] demonstrated that the rate of permeation from a vehicle was dependent on the interaction between the drug, vehicle affinity, and/or vehicle–skin effects, as shown by the enhanced permeation of solutes from oil-based vehicles [26]. Percutaneous absorption studies [19,42] on topical corticosteroids using the VCA have also demonstrated the effect of vehicle–drug interactions by showing the effects of different formulation vehicles on the topical bioavailability and potency of drugs [42,103]. Queille-Roussel et al. [104] demonstrated differences in the potency of calcipotriol and betamethasone dipropionate combination, where the potency was higher in the cutaneous foam compared to the ointment formulation [104]. Other physicochemical properties, such as hydrogen bonding compatibility between the vehicle and solute, may also be used to enhance drug permeation by increasing drug solubility [102]. Hattori et al. [105] demonstrated an increase in the solubility of nobiletin (drug) using choline and geranic acid (vehicle), as a result of hydrogen bonding between the drug and vehicle, which, in turn, enhanced transdermal absorption [105].

### 3.2. Skin–Vehicle Interactions

**Figure 4:**
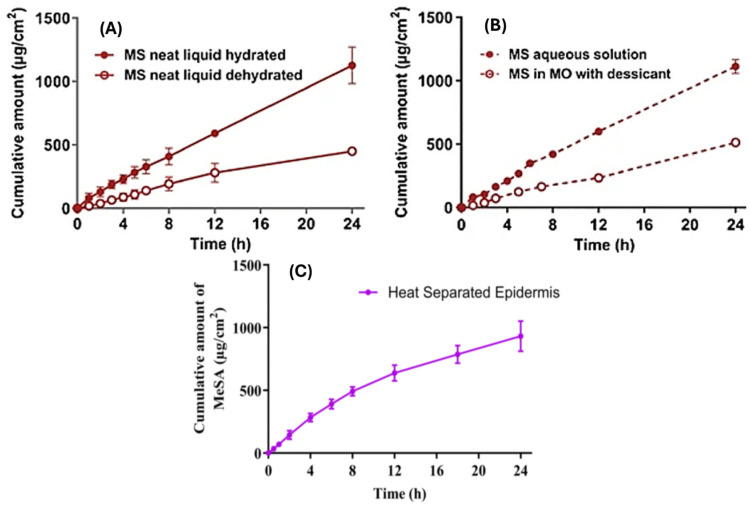
In vitro cumulative amount permeated versus time of: (**A**) neat (pure) Methyl salicylate (MS) ester through human epidermal membranes under hydrated (closed symbols) and dehydrated (open symbols) conditions (mean ± SD, *n* = 4–5) [109]; (**B**) MS ester through human epidermal membranes from saturated aqueous solutions (hydrated; closed symbols) and in mineral oil (MO) with desiccant (dehydrated; open symbols) (mean ± SD, *n* = 4–5) [109]; (**C**) MS easter in Metsal™ Cream (a topical product) applied on human epidermal membranes (mean ± SEM, *n* = 3) [110].

The physicochemical properties of a pharmaceutical vehicle can affect the integrity and elasticity of the skin barrier and the diffusion pattern, which, in turn, affects the rate of permeation [21,98]. During percutaneous absorption, skin–vehicle interactions contribute notably to regulating the degree of permeation by altering the diffusional resistance of the SC and the extent of hydration, thereby altering the degree of permeation [91,97]. The state of hydration of the skin is a significant factor in skin permeation. Hydration is a result of water diffusion from the epidermis, and it promotes drug diffusion through the skin, which can be modified by different formulation vehicles or the use of occlusive dressings [106]. Generally, ointments retain a higher degree of water and increase hydration compared to less occlusive water-in-oil emulsions [98]. However, to an extent, the hydration of the SC is relative to the environmental humidity; therefore, a water gradient exists within the SC [27]. Wurster and Kramer [107] demonstrated an increase in the steady-state permeability of salicylate esters with an increase in the hydration of the skin [108]. The water activity (a_w_) of a vehicle, representing the thermodynamic energy status of water in the formulation vehicle, has been shown to be an important parameter that influences percutaneous absorption. Angamuthu et al. [108] demonstrated the correlation between a_w_ and permeation, shown by a decrease in vitro permeation of caffeine relative to a decrease in a_w_ of the vehicle. The degree of drug permeation was also shown to be related to the skin’s hydrodynamics and structural changes [108]. Figure 4 demonstrates the skin permeation of methyl salicylate across epidermal membranes under three different conditions. First, as a pure liquid at room temperature, the permeability of methyl salicylate can be studied as a pure drug (no vehicle, hence no interaction of the skin with a vehicle). The second scenario is where an aqueous solution of methyl salicylate is studied. In both scenarios, steady-state flux was observed to be similar under hydrated and dehydrated conditions [109]. We found a third scenario, where methyl salicylate penetration from METSAL cream (containing 28.3% methyl salicylate) was studied under similar conditions and using the same human skin preparation (heat separated human epidermal membranes). Although steady-state flux was not quoted in the original publication [110], it can be observed that the cream vehicle with its oil phase, which has close to 45 times higher saturated solubility compared to aqueous solubility [109], led to a higher inital flux up to 8 h, after which the flux was reduced. This demonstrates the impact of both enhanced solubility as well as vehicle interactions with the skin.

**Figure 5:**
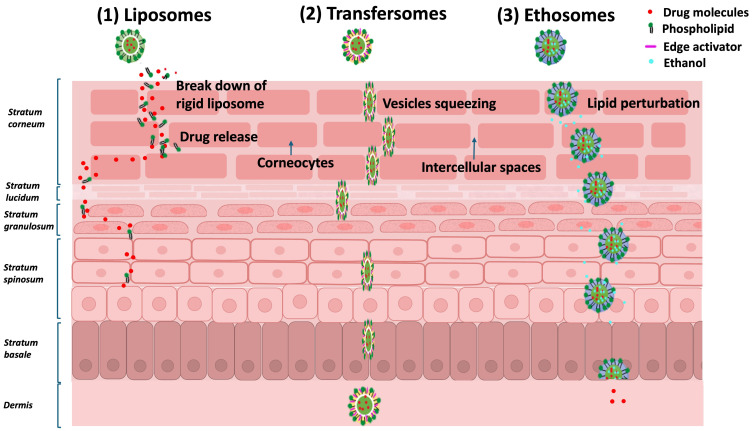
Diagrammatic representation of lipid nanocarriers, including (1) liposomes, which release drugs upon the breakdown of their lipid bilayers; (2) transfersomes, incorporating edge activators that deform and pass through intercellular spaces across multiple skin layers to deliver drugs into the dermis; and (3) ethosomes, containing ethanol as a permeation enhancer, which disrupts lipid organization and facilitates deep penetration through the skin [115,121].

Alcohols are often used as co-solvents and enhancers to increase the solubility of lipophilic compounds in aqueous vehicles which, in turn, increases the partitioning of the drug from the vehicle into the SC [64,65]. Short-chain aliphatic alcohols are generally considered to improve skin permeation [111]. Solvents, such as propylene glycol and ethanol, alter the chemical properties of the SC, which increases the partitioning of the drug as well as the solubility within the SC [65]. By comparing the effect of different alcohol-based vehicles (methanol, ethanol, and isopropanol solutions) on skin permeability, conducted in a Franz cell using porcine skin, Ossowicz et al. [111] demonstrated the enhanced permeation effect and skin retention of ibuprofen modified with L-valine alkyl esters, from the high solvent effect of alcohols. Propanol was shown to have the greatest effect on increasing the permeability of ibuprofen, and permeability was demonstrated to increase from an increase in the chain length of the alcohol [111], therefore, in skin permeation, the preferred reference state is a saturated solution, as this state can be used as a vehicle to enhance transdermal absorption. An earlier study by Roberts and Anderson [97] also demonstrated the effect of skin-vehicle interactions on the degree of phenol permeability. The skin-vehicle interactions altered the diffusional resistance of the SC as well as the extent of hydration, thereby altering the degree of permeation [97]. A significant vehicle effect on the degree of penetration of benzoic acid and butenafine hydrochloride in the SC was observed by Zhang et al., [64] with different vehicles of ethanol, isopropyl alcohol, and isopropyl myristate, resulting in different degrees of permeation [64]. Skin-vehicle interactions have also been used in the design of lipid nanocarriers e.g., liposomes (phospholipid vesicles), transfersomes (phospholipid-based vesicles with an edge activator) [112,113,114], and ethosomes (phospholipid-based vesicle with ethanol) [115,116], that are intended to transport hydrophilic and lipophilic molecules [117,118,119]. They are made up of drugs dispersed in lipid vehicles that interact with the SC lipids and enhance permeation (Figure 5) [120].

### 3.3. Skin–Drug Interactions

The physicochemical properties of the drug, such as molecular weight, structure, and lipophilicity, can facilitate percutaneous absorption by altering skin conditions [122]. Based on the Dalton 500 rule, permeation through the SC is more likely for molecules with a molecular weight of less than 500 Da; hence smaller molecules can penetrate easier compared to larger molecules [123]. The Stokes–Einstein equation also suggests that smaller molecules have a higher permeability compared to larger ones, which can be mathematically represented as follows:

(9) $$D=\frac{RT}{6\pi \eta rN}$$

where D is the diffusion coefficient of drug, R is the molar gas constant, T is the absolute temperature, N is Avogadro’s number, r is the spherical radius of the drug, and η is the membrane viscosity [61]. The smaller the radius of the drug, the greater the diffusion coefficient.

The molecular structure of the drug also plays a significant role in drug diffusion across the skin, which affects bioavailability [20]. The hydrogen bonding potential of the molecules determines the extent of binding of the drug to the skin [20]. The temperature of the skin also holds a considerable value in drug delivery by altering the barrier of the SC and the release of a drug from a formulation vehicle. Heat exposure can increase drug permeation, which is related to an increase in drug diffusion, partitioning, and solubility of the drug in the SC [124]. The solubility of the drug in the SC also increases due to dilation of skin penetration pathways from temperature changes, which increases absorption [125]. A study conducted by La Count and associates [126], using a combined computational model and experimental data to evaluate heat effects on the transdermal delivery of nicotine via exercised human cadaver skin using in vitro (modified Franz diffusion cell) and in silico models (computational model), demonstrated a two-fold increase in nicotine flux at 42 °C compared to when the temperature was 32 °C. It was observed from the study that increasing skin surface temperature increased the skin permeation of nicotine, with highest flux found during early heat application [126].

Solvent deposition is often used in the assessment of dermal absorption, where solids precipitate after solvent evaporation [18,127], as demonstrated by Yu et al. [127] in a study on the absorption of solvent-deposited niacinamide and methyl nicotinate on ex vivo human skin. Their study used a mechanistic transient diffusion model to illustrate the difference in rates of absorption of the two drugs, niacinamide and methyl nicotinate, because of different release rates from the deposited solid. They demonstrated evidence of the effect of skin–drug interactions shown by an increase in the permeability of methyl nicotinate as a result of a potential disruption to the lipid structure in the SC barrier by the drug [127]. Skin–drug interactions have also been used in the design of film-forming systems, which improve adherence and prolong the contact time between the drug and skin, thereby promoting drug absorption. They are formulated in a volatile vehicle that evaporates after application, leaving behind a film containing excipients and the drug in a supersaturated state. This increases the thermodynamic activity of the drug without disrupting the SC, which, in turn, enhances drug permeation [128,129]. Prolonged contact time between the drug and skin in a film-forming system has been demonstrated to increase drug permeation of poly(lactic-co-glycolic acid) (PLGA) [130]. Chemical sunscreens are another example of skin–drug interaction. These sunscreens need to be absorbed into the skin before activation. Activation, or the process of absorbing UV radiation and releasing heat, occurs in the SC [131].

### 3.4. Metamorphosis of Topical Vehicles—Complex Drug–Skin–Vehicle Effects

**Figure 6:**
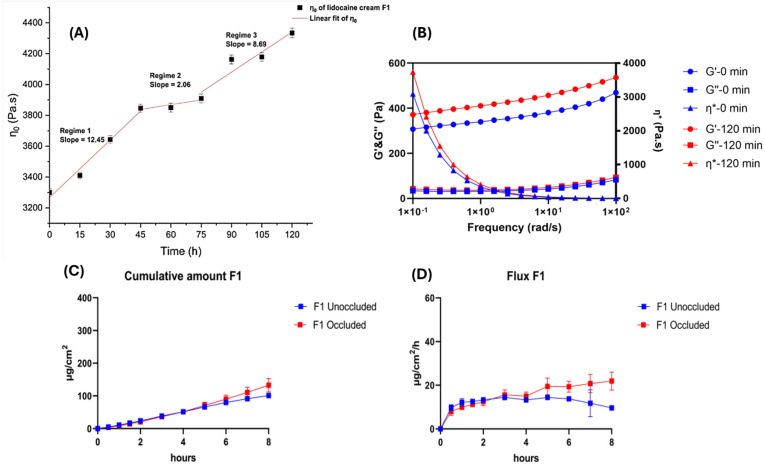
(**A**) Evaporation/time dependence of the zero-shear viscosity (*η*0) of O/W lidocaine cream F1. (**B**) Frequency sweep of O/W lidocaine cream F1 samples at 0 and 120 min (G: elastic modulus; G″: viscous modulus; η*: complex viscosity). In vitro permeation profiles of lidocaine in O/W lidocaine cream F1: (**C**) cumulative amount of lidocaine in F1 under unoccluded and occluded conditions, and (**D**) flux of lidocaine in F1 under unoccluded and occluded conditions [32].

Vehicles are three-dimensional structural matrices, considered to be monophasic (e.g., ointment), biphasic (e.g., cream), or tri/multiphasic (e.g., cream paste), designed for drug incorporation [132]. Metamorphosis (change in composition and microstructure) of formulation vehicles has been shown to increase the concentration of the API on the skin, which leads to the supersaturation of the formulation and increased thermodynamic activity of the drug, which, in turn, can raise the degree of drug permeation [32,88,133]. Vehicle metamorphosis occurs when volatile solvents present in dermal formulations evaporate after product application onto the skin, leaving a modified formulation with increased drug concentration [132]. Ethanol and propylene glycol are commonly used volatile solvents in topical formulations [88] that have been demonstrated to increase the rate of drug release from the formulation through vehicle metamorphosis [37]. The rate of solvent evaporation is dependent on the concentration of volatile solvent in the formulation, demonstrated by the rapid evaporation of gel formulations, which are composed of a higher alcohol and water content, compared to that of creams [88]. A topical system that has also been designed using vehicle metamorphosis is medicated foams [134,135]. After the solvent evaporation of volatile substances, the remaining drug may precipitate or dissolve in the remaining vehicle, forming a supersaturated system that increases the degree of drug permeation [136,137]. The U.S. Food and Drug Administration [90] has approved a topical foam containing 4% minocycline for the treatment of acne vulgaris. Research on this formulation and profile of 4% minocycline topical foam has shown a high concentration of minocycline in sebaceous appendages, with minimal systemic exposure of 730 to 765 times lower than that of oral minocycline, and a reduced risk of minocycline degradation [138]. Another example is tahe film-forming system designed for the delivery of a nitroimidazole compound, which is prepared as a solution but metamorphoses into a film after solvent evaporation, to increase the contact time of the drug and skin surface, thereby increasing the degree of drug permeation [139]. In research by Jin et al. [32], metamorphosis of lidocaine cream under “in use” conditions was observed (Figure 6). The viscosity and elastic modulus of the prepared sample increased with the time of evaporation, which may be related to the aggregation of carbopol micelles as well as the crystallization of the API. There was also a decrease (by 32.4%) in the permeability of lidocaine for the formulation (2.5% lidocaine) in unoccluded cells as compared to occluded cells [32].

## 4. Conclusions, Future Perspectives, and Limitations

This review provides an analysis of the pharmacokinetic mechanisms involved in percutaneous absorption, encompassing drug release from a vehicle, partition into and diffusion of the drug within the SC, subsequent diffusion of the drug into the viable epidermis, and absorption and distribution of the drug into the systemic circulation. By targeting specific pharmacokinetic phases, novel skin drug delivery systems, such as film-forming systems and modified foams, which have demonstrated increased drug absorption rates, have been developed. This review also discusses the role played by solute–vehicle–skin interactions in altering the degree of drug absorption through modifications of the drug and vehicle’s physicochemical properties. Understanding pharmacokinetic mechanisms and elucidating drug–vehicle–skin interactions involved in the percutaneous absorption of drugs aid in the development and optimization of novel topical and transdermal delivery systems. Limitations in the use of the available pharmacokinetic techniques are prevalent despite constant developments. Sampling directly within the skin, for example, is still a challenge. Recent topographic and microperfusion-based sampling techniques are capable of overcoming these in the future.

Enhanced knowledge and understanding in recent years, however, has ensured that topical and transdermal delivery is better today than at any point in history. Advanced strategies, including applications of nanocarriers combined with physical methods such as ultrasound, laser, and microneedles, have demonstrated the potential to enhance the overall therapeutic efficacy of transdermal drugs. However, human skin is highly sensitive and has a complex structure together with different biophysical characteristics. Establishing a unified system for a wide range of APIs loaded into novel nanocarriers is thus necessary to control interactions among drugs, skin, and carriers/vehicles. This approach can minimize risks as well as provide more effective pharmaceutical therapies, with positive impacts on patient compliance. Additionally, considering the impact of age and skin conditions, along with investing time and resources in nanotechnology, biomaterials, and formulation development, can lead to significant advances in topical and transdermal delivery systems.

## Data Availability

This article reviews the existing literature. All data discussed are available from the original publications cited within the article.
